# Characterization of the Volatile Components of Essential Oils of Selected Plants in Kenya

**DOI:** 10.1155/2020/8861798

**Published:** 2020-12-15

**Authors:** Lydia G. Mugao, Bernard M. Gichimu, Phyllis W. Muturi, Simon T. Mukono

**Affiliations:** ^1^Department of Agricultural Resource Management, University of Embu, P.O. Box 6—60100, Embu, Kenya; ^2^Department of Physical Sciences, University of Embu, P.O. Box 6—60100, Embu, Kenya

## Abstract

Essential oils are secondary metabolites that plants produce for protection from pests and predators, attraction of pollinators, and seed dispersal. The oils are made up of a mixture of compounds that give a characteristic flavour and odour. Currently, essential oils are receiving great attention in research for their phytochemical and antimicrobial activities. However, there is scanty information on the chemical composition of many plants. This study provides a detailed analysis of the chemical composition of essential oils of ginger, garlic, tick berry, and Mexican marigold in Kenya. The essential oils were extracted by steam distillation and analysed by gas chromatography–mass spectrometry. The study identified a total of 52 different chemical classes from the essential oils of the four different plants that were analysed. Their percentage composition was also found to vary between the test plants. The essential oils of Mexican marigold constituted the highest composition of the identified chemical classes at 71.2%, followed by ginger at 55.8%, while both tick berry and garlic oils constituted 53.8% of the total classes identified. Terpenes constituted the highest composition in the essential oils of all the four test plants. Other major chemical classes included esters, ketones, organosulfurs, alkanes, cycloalkanes, steroids, aromatic hydrocarbons, and alkanols. Some of these chemical compounds have been shown to have a huge utility potential in biopesticides, pharmaceutical, and food industries, and hence, their industrial extraction and purification from the essential oils of these plants are recommended.

## 1. Introduction

Essential oils are secondary metabolites that plants produce for protection from pests and predators, attraction of pollinators, or seed dispersal [1]. The oils are located in different parts of the plant such as roots, stems, leaves, flowers, fruits, and even in seeds depending on the plant species [2]. In these plant parts, the essential oil is accumulated in cells, secretory cavities, or glandular hairs [3]. Almost every part of a plant can produce essential oil, which can be extracted and exploited in various industrial ways [4]. The essential oils are highly volatile [5], transparent, and lipid-soluble liquids [6]. They are soluble in organic solvents such as benzene, toluene, acetone, ethanol, and methanol that have less density compared with water [7].

Essential oils are composed of a complex mixture of compounds which give a characteristic odour and flavour to the plants [1]. The compounds are grouped into main compounds (20–95%), secondary compounds (1–20%), and trace compounds with less than 1% concentration [8]. The flavour and aroma of plants are influenced by the type and amount of compounds present in the essential oil [9, 10]. Oxygenated monoterpenes are the major contributor to the taste and aroma of essential oils [11]. Essential oils from plants such as lemon grass have been used for a long time in aromatherapy and medicinal applications [4]. There are other compounds that bring about variation in the colour of essential oils from different plants [4]. Most essential oils contain natural monoterpenes and sequiterpenes, with a variety of functional groups that contribute to antifungal and antibacterial properties [12].

The essential oils of similar plants have been reported to have differences in composition depending on the geographical region that the plant is growing [13]. For example, Raina et al. [14] reported that ginger oil composition varied with the geographical origin of the plant. According to Fakim et al. [15], zingiberene was found to vary in quantity from different countries where ginger was growing. In India, zingiberene quantity in the essential oil was 46.2%, 38.12% in China, 29.0% in Nigeria, 20–28% in Australia, 9.5% in Mauritius, 3.6% in Central African Republic, and 1.5% in Srilanka. Different growth stages of the plants also bring about variations in the essential oil composition within the same organ of the plant [16].

There are different methods of essential oil extraction such as steam distillation and low temperature distillation for those compounds that require low temperatures [2] and hydrodistillation and extraction with solvents [17]. The method of extraction determines the quality of essential oil. Inappropriate extraction procedures can lead to damage or change of the chemical nature of the essential oil [4]. Many natural products are thermally unstable and can easily be damaged during thermal extraction [18]. The method of extraction used depends on the kind of compounds present in the oil and the location of the oils within the vegetative structure of the plant species [17].

Steam distillation method has been used widely for commercial extraction of essential oils [19]. However, it is difficult to control the heat transfer throughout the extraction process and the extraction time using steam distillation [4]. In addition, steam distillation extracts up to 93% of the essential oil [20]. In order to increase the content of essential oils extracted, this study utilized a new design of steam distillation developed by Masango [20] that reduces the loss of polar compounds in waste water. The plant material is placed above the water such that it is not in contact with the water, but the steam from heated water passes through it and extracts water-soluble compounds, which are then dissolved in the aqueous fraction at a lower extent [20]. Alternatively, Gas Chromatography and Mass Spectrometry (GC-MS) is one of the best tools to identify and quantify the constituents of essential oils because of its simplicity, rapidity, accuracy, and efficiency [21]. Consequently, GC-MS has of late been widely used in chromatographic fingerprinting of medicinal plants [22–24].

Bioactive compounds present in essential oils have been found to act as biopesticides locally, but they are not adequately utilized by commercial industries. Alternatively, herbal plants that produce essential oils have been utilized as remedies of various problems without knowledge of their quality or chemical composition [25] though with lesser side effects [26]. It is therefore necessary to analyse the composition of the volatile components of essential oils of different plants for the purpose of separating individual compounds that may have different uses in different industries. This study was aimed at extracting essential oils from garlic (*Allium sativum*), ginger (*Zingiber officinale*), Mexican marigold (*Tagetes minuta*), and tick berry (*Lantana camara*) by steam distillation and analysing their chemical composition using the GC-MS method.

## 2. Materials and Methods

### 2.1. Collection of Plant Materials

Fresh tick berry and Mexican marigold leaves were collected from the field at the University of Embu farm in the year 2019, while ginger rhizomes and garlic bulbs were obtained from the local open air market. One kilogram each of tick berry and Mexican marigold leaves were washed under tap water, rinsed in three changes of distilled water, and then air-dried in the laboratory at room temperature (25°C) to evaporate the moisture content. One kilogram each of garlic cloves and ginger rhizomes were also washed with tap water and then rinsed with three changes of distilled water. The test materials were also air-dried in the laboratory and later peeled to remove the outer coverings. All fresh test materials were stored in the laboratory under room temperature awaiting extraction of essential oils.

### 2.2. Extraction of Essential Oils

Extraction of essential oils was done following a modified procedure described by Adams [27] and adopted by Mugao et al. [28]. Vertical steam distillation unit, consisting of a hot plate, boiling flask, biomass flask, still head, condenser, and receiving flask (separating funnel), was used for dry steam distillation of plant material. The fresh materials of each plant were put separately in the biomass flask, and the distillation unit was switched on. Steam was produced in the boiling flask by heating distilled water with the hot plate. The steam moved upward into the biomass flask where essential oils and other water-soluble plant compounds were moved as vapour through the still head, condensed in the water-cooled condenser, and collected in the receiving flask (separating funnel). The receiving flask separated the heavier-than-water oils from the lighter-than-water oils while allowing excess water-soluble compounds to be drained out and collected separately. The obtained essential oil samples were dried to remove any water molecules by passing them in anhydrous sodium sulphate (Na_2_SO_4_) and then stored in sealed vials at 4°C awaiting the GC-MS analysis of the chemical compounds.

### 2.3. Analysis of the Volatile Components of Essential Oils

Gas chromatograph (GC) Agilent 7890B with autosampler was used to analyse the composition of the volatile components of essential oils extracted from ginger, garlic, tick berry, and Mexican marigold. Mass spectrometry (MS) detector Agilent 5977A was an integral part of the gas chromatograph. The GC-MS records were processed by means of GC-MS ChemStation software. The GC was equipped with a mass selective detector (MSD), an autosampler, and a highly polar DB-wax capillary column (length 30.0 mx; inner diameter 0.25 mmx; inner film thickness 0.25 *μ*m) with 100% polyethylene glycol stationery phase.

The analysis followed the modified procedure described by Omolo et al. [29]. The analysis started after 3.95 minutes (solvent delay). The GC condition had an initial oven temperature of 40°C maintained for 3 minutes. The temperature was then gradually increased with gradients of 10°C/minute up to 60°C, 5°C/minute up to 90°C, and 4°C/minute up to 250°C and maintained there for 8 minutes. Subsequent GC working conditions utilized hydrogen gas with a constant flow of 1 ml/minute as the carrier gas and n-hexane as matrix solvent. The MS working conditions comprised of an ion source and transfer line temperature maintained at 300°C, a regime of injection split in a ratio of 15 : 1, a split flow of 22.5 mL/minute, and a mass range of 20–300 m/z. The injection was done with a 10 *μ*l injection syringe containing 1 *μ*l of injection volume at 250°C injection temperature.

The composition of the essential oils was eluted at different retention times, identified, and obtained by the HES Auto Tune (HES ATUNE.U) by scan. The chromatogram obtained was analysed, and each peak was checked by determining the percent area on the chromatogram, retention time, spectrum, and base peak and then referring to the characteristic mass spectra of compounds listed in the National Institute of standards and Technologies (NIST) using the software of Agilent Topi [30]. Their percentage relative abundances were calculated using

(1) $$\begin{array}{c} \%\text{relative abundance}=\frac{\text{peak area of the document}}{\text{Total sum of all peak areas}}\times 100. \end{array}$$

The compounds were then classified into different classes on the basis of presence of heteroatoms or the functional groups of the molecule.

## 3. Results and Discussion

### 3.1. Chemical Classes Identified from Essential Oils

The study identified a total of 52 different chemical classes from the essential oils of the four different plants that were analysed. Their percentage composition was also found to vary between the test plants (Table 1). The essential oils of Mexican marigold constituted the highest composition of the identified chemical classes at 71.2%, followed by ginger at 55.8%, while both tick berry and garlic oils constituted 53.8% of the total classes identified.

There were 18 major classes that were identified with average percent chemical composition of >1% as shown in Table 1. Terpenes constituted the highest composition in the essential oils of all the tested plants. These results also corroborate with those of Swamy et al. [31] and Matos et al. [32], who reported that terpenes constitute about 50–95% of the essential oils. Dhifi et al. [33] also reported that terpenes constitute the majority of chemical classes in essential oils of various plants. Murugesan et al. [34] also revealed that tick berry essential oils from Tamil Nadu region in India had more terpenes than other compounds. Other major chemical classes identified in this study included esters, ketones, organosulfurs, alkanes, cycloalkanes, steroids, aromatic hydrocarbons, alkanols, cycloalkanols, alkenols, carbonates, fatty acids, carbaldehyde, aldehydes, alkenes, ethers, carboxylic acid, alkaloids, and organic acids listed in order of abundance (Table 1). The listed classes were present in relatively higher abundance either in all or at least three of the tested plants. Dhifi et al. [33] also reported that, apart from terpenes, essential oils also contain various chemical classes including alcohols, ethers, aldehydes, ketones, esters, amines, amides, and phenols.

The percent composition of the different chemical classes in the essential oils of the tested plants was found to vary among the test plants. Ginger essential oil had the highest amount of terpenes (42.18%), followed by garlic (39.57%), tick berry (37.26%), and Mexican marigold (20.94%). Although ginger and garlic, which are majorly spices, were found to contain high levels of terpenes, the compound reportedly contributed less to flavour and fragrance of the essential oils [31, 32]. Mexican marigold had the highest amount of esters (14.01%) and relatively high amount of ketones (8.93%) and alkanols (4.73%). Ginger had the highest composition of ketones (8.99%) and fatty acids (3.67%) and relatively high amount of alkanes (7.42%) and cycloalkanols (4.13%). Garlic recorded the highest composition of organosulfur (15.01%), aromatic hydrocarbons (6.36%), carbonates (3.71%), and carbaldehyde (3.85%). Garlic also had a relatively high amount of fatty acids (3.61%). Tick berry had the highest composition of alkanes (7.55%), cycloalkane (7.31), steroid (7.08%), alkanols (5.12%), alkenols (5.73%), and aldehydes (4.67%) as shown in Table 1. The composition of other minor compounds with average composition of <1% also varied among the test plants and were found to be present in either three, two, or one out of the four plants that were analysed except organic acids and phthalates, which were present in all the plants.

### 3.2. Specific Compounds Identified from Essential Oils

Further analysis of the identified chemical classes showed that their specific composition varied among the test plants.  Table 2 shows the composition of the five major specific compounds in each of the four test plants. This study identified bicyclogermacrene and 3-carene (terpenes), cholesterol (steroid), (1R, 7S, E)-7-Isopropyl-4,10-dimethylenecyclodec-5-enol (alkenol), and bicyclo[5.2.0]nonane,2-methylene-4,8,8-trimethyl-4-vinyl-(cycloalkane) as the major compounds in tick berry (Table 2). Contrary to this observation, the essential oils obtained from the leaves and flowers of tick berry in Cameroon and Madagascar contained curcumene (24.7%), ᵦ-caryophyllene (13.3%), and davanone (15.9%) as the major compounds [35]. The first two are terpenes, while the third one is a ketone. In our study, curcumene was not detected in tick berry, while caryophyllene and davanone were available at relatively low amounts of 0.16% and 0.13%, respectively. Silva et al. [36] also reported that essential oil of tick berry from north Brazil contained limonene, *α*-phellandrene, germacrene D, and zingiberene as the major compounds. We also identified *α*-phellandrene and germacrene D in tick berry essential oil but no zingiberene and limonene. Bicyclogermacrene, germacrene D, and *β*-caryophyllene have been reported as chemical markers of lantana species [37]. However, we found bicyclogermacrene and *β*-caryophyllene to be present also in the other tested plants, while only 0.20% of germacrene D was detected.

The major compounds identified in the Mexican marigold essential oil in this study were chrysantenyl 2-methuylbutanoate (3.07%), which is an ester, papaveroline, 1,2,3,4-tetrahydro-3-O-methyl- (3.02%), which was an alkaloid, 3,5-dimethylanisole (3.01%), which is an aromatic hydrocarbon, t-butylhydroquinone (2.96%), which is a hydroquinone, and dodecanoic acid (2.85%), which is an ester (Table 2). This finding was a departure from the reports of Marotti et al. [38] and Singh et al. [39], who reported that Mexican marigold essential oil obtained from aerial plant parts contained major compounds such as limonene, (Z)-*β*-ocimene, (E)-*β*-ocimene dihydrotagetone, (Z)- and (E)-tagetones, and (Z)- and (E)-tagetonones. However, our study also identified (E)- and (Z)-targetones, dihydrotagetone, and (E)- and (Z)-tagetonones in Mexican marigold essential oil but were not the major compounds.

The major compound in ginger essential oil was corymbolone (3.59%) belonging to the family of ketones, followed by zingiberene (3.54%) and bicyclogermacrene (3.49%), which is a terpene. Others were 4,4-dimethyl octane (3.21%), which is an alkane, and cis-2-norbornanol (3.08), which is a cycloalkanol (Table 2). These findings partially corroborate with some earlier reports from other studies and at the same time contradicte other reported findings. For example, zingiberene was reported as the major constituent of ginger oil in India [14] and Mauritius [15]. However, hydro-distilled ginger essential oil from a 10-month-old ginger rhizome cultivated in North east India did not contain any zingiberene [40]. Agrawal et al. [41] found curcumene to be the major constituent of steam-distilled ginger oil in India, while citral was reported as the major constituent of ginger oil in Central Africa Republic [42]. In our study, only 1.13% of *α*-curcumene and 0.47% of citral was detected in ginger. Zingiberene was also present in garlic though in relatively smaller quantities.

In garlic, the major compound was identified as allyl(Z)-prop-1-enyl trisulfide (4.30%), which is an organosulfur, followed by 2-((2R,4aR,8aS)-4a-methyl-8-methylenedecahydronaphthalen-2-yl)-prop-2-en-1-ol (3.93%), which is a terpene, and longifolenaldehyde (3.86%), which is a carbaldehyde. Others were nerolidol (3.63%), which is a terpene and (E)-1-methyl-2-(prop-1-en-1-yl) disulfane (3.40%), which is an organosulfur (Table 2). These findings did not agree with those of Rao et al. [43] who reported that terpenes were rare in garlic essential oil, and in most cases, only little amounts of monoterpene hydrocarbons such as sabinene and limonene are present. In our study, two terpene compounds were among the major compounds identified in garlic oil, while sabinene and limonene were not detected at all. In fact, our study identified a total of 37 terpenes in garlic making them the most abundant compounds followed by organosulfurs (21) and esters (18). This observation thus contradicted that of Rao et al. [43] and Abu-Lafi et al. [44] who reported that garlic oil had more sulfur compounds than terpenes. The high detection of terpenes in garlic essential oils in our study can be attributed to fresh extraction. Drying of garlic before extraction of essential oil has been reported as one major cause of loss of terpenes from the essential oil [45].

The major organosulfurs that were identified in this study were similar to those identified by other researchers [45–49]. They included allyl(Z)-prop-1-enyl-trisulfide (4.30%), (E)-1-methyl-2-(prop-1-en-1-yl) disulfane (3.40%), and diallylsulphide (2.94%) among many others. The organosulfur compounds are said to be antifungal, antibacterial, anti-inflammatory, antioxidant, anticarcinogenic, antidiabetic, cardioprotective, and neuroprotective [46, 50, 51]. Therefore, garlic is a high value crop with potentially high utility in medical, agrochemical, and food industries. Roberta and Guido [52] also identified E-E farnesal in garlic essential oil, but in our study, this compound was only found in ginger essential oil.

The varying reports of the volatile composition of the same plants from the findings of other previous studies conducted in different countries were attributed to differences in geographical location which affected the agroecological conditions and hence the biochemical composition [37, 53, 54]. Other causes of such diversity may include the nature of chemotypes [55, 56], harvesting stage of the tested plants, plant parts from which the oil is extracted [54, 57, 58], soil type and its nutrient status [37, 57], climatic conditions under which the plants were grown, method of harvesting, and oil extraction method [59–61]. The time of the year that the plant material was harvested to extract the oil may also determine the concentration of the essential oil compounds [62, 63]. In addition, the differences in chemical composition of essential oil from the same plant part could be influenced by environmental (climate and soil factors) [64, 65], developmental, and genetic factors [58, 66].

The study further revealed the most common compounds that were present in the essential oils of all the tested plants as shown in Table 3. The majority were terpenes (5), followed by alkanes (2), while carbaldehydes, phthalates, and esters had one compound each that was common in all the four plants. The most abundant terpenes included bicyclogermacrene, 3- careen, and citral, which were most abundant in tick berry (Table 3). The only ester that was found in all the test plants was *γ*-hexalactone (Table 2). The findings of this study therefore corroborate with earlier reports from other studies that state that terpenes are by far the most dominant constituents of essential oils [67–69].

## 4. Conclusion and Recommendations

It can be concluded that the chemical composition of the essential oils of ginger, garlic, tick berry, and Mexican marigold has a remarkable variation attributable to the species' genetic variation and probably the ecological conditions under which these plants are grown. Some of the compounds identified in this study such as terpenes have unutilized potential in biopesticides, pharmaceutical, and food industries. Therefore, industrial extraction and purification of the essential oils in these plants is recommended. The four tested plants were found to have some common chemical compounds and can therefore be exploited for synergistic utility.

## Figures and Tables

**Table 1:** Composition of chemical classes identified from different essential oils.

S/no.	Chemical class	Percent composition				Average
		Tick berry	Mexican marigold	Ginger	Garlic	
1	Terpenes	37.26	20.94	42.18	39.57	34.99
2	Ester	5.02	14.01	5.8	4.58	7.35
3	Ketones	2.07	8.93	8.99	4.61	6.15
4	Organosulfur	5.19	1.79	0.04	15.01	5.51
5	Alkanes	7.55	0.87	7.42	3.20	4.76
6	Cycloalkane	7.31	5.82	4.99	0.12	4.56
7	Steroid	7.08	2.01	2.26	4.82	4.04
8	Aromatic hydrocarbon	0.73	4.16	4.37	6.36	3.91
9	Alkanols	5.12	4.73	2.51	0.62	3.25
10	Cycloalkanol	4.04	1.55	4.13		2.43
11	Alkenols	5.73	0.54	2.73	0.59	2.40
12	Carbonates		2.12	1.61	3.71	1.86
13	Fatty acids			3.67	3.61	1.82
14	Carbaldehyde	0.59	2.41	0.28	3.85	1.78
15	Aldehydes	4.67	0.67	1.3		1.66
16	Alkenes	0.12	5.32	0.48		1.48
17	Ethers	0.07	2.56		2.53	1.29
18	Organic acid	0.98	3.52	0.24	0.26	1.25
19	Carboxylic acid		2.81	1.12	0.32	1.06
20	Alkaloids	0.09	3.50		0.06	0.91
21	Heterocyclic hydrocarbon	0.03	0.43		2.49	0.74
22	Hydroquinones		2.95			0.74
23	Cyclic carboxylic ester (lactone)		2.25			0.56
24	Nitrile		0.15		1.81	0.49
25	Furans	1.28	0.09	0.47		0.46
26	Benzyl carbazate	1.67	0.09			0.44
27	Dione	1.71	0.02			0.43
28	Amide	0.93		0.5	0.16	0.40
29	Cycloketone (cyclohexanone)			1.14		0.29
30	Antihistamine		1.12			0.28
31	Thiozole			1.05		0.26
32	Polyphenol			1.04		0.26
33	Hydroxysteroid				0.78	0.20
34	Phthalate	0.11	0.06	0.51	0.04	0.18
35	Silane		0.6		0.07	0.17
36	Aromatic hydrocarbon (toluene)		0.64			0.16
37	Amine	0.03	0.39		0.08	0.13
38	Chloroalkyne		0.45			0.11
39	Cycloalkenol			0.07	0.26	0.08
40	Thiophene	0.33				0.08
41	Aromatic amine (aniline)			0.29		0.07
42	Phenanthrenol		0.24			0.06
43	Allethrin			0.21		0.05
44	Phenol	0.17				0.04
45	Corticosteroid		0.15			0.04
46	Polycylic aromatic hydrocarbon				0.12	0.03
47	Dye		0.07			0.02
48	Bithiophene		0.07			0.02
49	Pyrrolidinophenone			0.04		0.01
50	Morpholine			0.04		0.01
	**Total compounds found**	**28 (53.8%)**	**37 (71.2%)**	**29 (55.8%)**	**28 (53.8%)**	

**Table 2:** Most abundant compounds in the major classes of volatile components.

S/no.	Chemical class	Individual compound	Tick berry	Mexican marigold	Ginger	Garlic
1	Terpene	Bicyclogermacrene	5.24	0.11	3.49	0.57
2	Steroid	Cholesterol	4.71	1.97		3.44
3	Terpene	3-Carene	4.36	0.39	2.89	0.08
4	Alkenol	(1R,7S,E)-7-Isopropyl-4, 10-dimethylenecyclodec-5-enol	3.96		2.74	0.60
5	Cycloalkane	Bicyclo[5.2.0]nonane,2-methylene-4,8,8-trimethyl-4-vinyl-	3.92		1.58	
6	Ester	Chrysantenyl 2-methuylbutanoate		3.07		
7	Alkaloid	Papaveroline, 1,2,3,4-tetrahydro-3-O-methyl-		3.02		
8	Aromatic hydrocarbon	3,5-Dimethylanisole		3.01		
9	Hydroquinone	t-Butylhydroquinone		2.96		
10	Ester	Dodecanoic acid, 1,2,3-propanetriyl ester		2.85		
11	Ketone	Corymbolone	0.53		3.59	0.86
12	Terpene	Zingiberene			3.55	0.32
13	Alkane	4,4-Dimethyl octane	3.34		3.21	0.04
14	Cycloalkanol	Cis-2-norbornanol			3.08	
15	Organosulfur	Allyl(Z)-prop-1-enyl trisulfide				4.30
16	Terpene	2-((2R,4aR,8aS)-4a-Methyl-8-methylenedecahydronaphthalen-2-yl)prop-2-en-1-ol		0.06	2.29	3.93
17	Carbaldehyde	Longifolenaldehyde	0.59	0.93	0.21	3.86
18	Terpene	Nerolidol			0.12	3.63
19	Organosulfur	(E)-1-Methyl-2-(prop-1-en-1-yl) disulfane				3.40

**Table 3:** Compounds that were present in all the tested plants.

S/no.	Chemical class	Individual compound	Tick berry	Mexican marigold	Ginger	Garlic
1	Terpene	Bicyclogermacrene	5.24	0.11	3.49	0.57
2	Terpene	3-Carene	4.36	0.39	2.89	0.08
3	Carbaldehyde	Longifolenaldehyde	0.59	0.93	0.21	3.86
4	Terpene	Bicyclo[2.2.1]heptan-2-one, 1,7,7-trimethyl-, (1S)-	0.15	2.66	0.14	1.88
5	Terpene	Citral	2.72	1.39	0.47	0.11
6	Terpene	Caryophyllene	0.16	0.05	0.64	0.35
7	Phthalate	Bis(2-ethylhexyl)phthalate	0.12	0.06	0.56	0.05
8	Alkane	Hexane,3-methyl-4-methylene-	0.05	0.35	0.04	0.06
9	Alkane	Dodecane, 1-fluoro-	0.17	0.23	0.02	0.04
10	Ester	*γ*-Hexalactone	0.05	0.04	0.05	0.05

## Data Availability

Most of the data used to support the findings of this study are included in the article. Additional data are available from the corresponding author upon request.
